# Patient and implant survival following joint replacement because of metastatic bone disease: A cross-sectional study of 130 patients with 140 joint replacements

**DOI:** 10.3109/17453674.2013.788437

**Published:** 2013-05-31

**Authors:** Michala S Sørensen, Kristine G Gregersen, Tomas Grum-Schwensen, Dorrit Hovgaard, Michael M Petersen

**Affiliations:** Department of Orthopedic Surgery, Copenhagen University Hospital, Rigshospitalet, Copenhagen, Denmark.

## Abstract

**Background:**

Patients suffering from a pathological fracture or painful bony lesion because of metastatic bone disease often benefit from a total joint replacement. However, these are large operations in patients who are often weak. We examined the patient survival and complication rates after total joint replacement as the treatment for bone metastasis or hematological diseases of the extremities.

**Patients and methods:**

130 patients (mean age 64 (30–85) years, 76 females) received 140 joint replacements due to skeletal metastases (n = 114) or hematological disease (n = 16) during the period 2003–2008. 21 replaced joints were located in the upper extremities and 119 in the lower extremities. Clinical and survival data were extracted from patient files and various registers.

**Results:**

The probability of patient survival was 51% (95% CI: 42–59) after 6 months, 39% (CI: 31–48) after 12 months, and 29% (CI: 21–37) after 24 months. The following surgical complications were seen (8 of which led to additional surgery): 2–5 hip dislocations (n = 8), deep infection (n = 3), peroneal palsy (n = 2), a shoulder prosthesis penetrating the skin (n = 1), and disassembly of an elbow prosthesis (n = 1). The probability of avoiding all kinds of surgery related to the implanted prosthesis was 94% (CI: 89–99) after 1 year and 92% (CI: 85–98) after 2 years.

**Conclusion:**

Joint replacement operations because of metastatic bone disease do not appear to have given a poorer rate of patient survival than other types of surgical treatment, and the reoperation rate was low.

Since the beginning of the 1990s, advances in the diagnosis and treatment of various types of cancer have led to a gradually increased survival in patients, and combined with the increase in the elderly population the number of patients suffering from metastatic bone disease has increased. Most of these patients can be treated without surgery, for example with radiotherapy, chemotherapy, bisphosphonates, or analgesic treatment. However, surgical treatment is a good treatment option for this group of patients in cases with a pathological fracture or impending fracture, and in patients with a solitary metastatic lesion (Rougraff 2000, Bauer 2005). Previous studies (Wedin et al. 1999, Harvey et al. 2012) have compared the failure rate after treatment of metastatic bone disease with a joint replacement to conventional osteosynthetic devices and shown a lower risk of failure of the bony reconstruction when a joint replacement was used. Patients suffering from bone metastases or hematological disease of bone often have relatively poor health, and in previous studies that have evaluated the probability of survival in this group of patients treated surgically for long bone or pelvic metastases, a 1-year survival of 16–70% was found (Wedin et al. 1999, Hansen et al. 2004, Camnasio et al. 2008, Harvey et al. 2012).

The aim of this study was 2-fold. Firstly, we wanted to determine the oncological outcome (survival) of patients who received an artificial joint replacement due to bone metastases or malignant hematological disease of the extremities, and secondly we wanted to determine what kinds of complications are seen in this group of patients (including an estimation of implant survival).

## Patients and methods

### Patients

In a cross-sectional study, we included all 130 patients (76 females) who received a joint replacement operation due to skeletal metastases (n = 114) or hematological disease (n = 16) from January 2003 through December 2008 at the Section for Tumor Surgery, Department of Orthopedic Surgery, Rigshospitalet, Copenhagen, Denmark (Table). The Section for Tumor Surgery is a tertiary referral center for orthopedic oncology surgery, which specializes in bone and soft tissue sarcomas and metastatic bone disease with major bone loss. Throughout the study period, it was departmental policy to prefer a joint replacement for treatment of an extremity metastatic lesion in proximity to a joint and to attempt a wide margin in solitary lesions. When hip replacement surgery was done, total hip replacement was preferred instead of a hemiarthroplasty. The patients received 140 joint replacements in total, and the mean age at the first operation was 64 (30–85) years (Table). Patients who received other orthopedic implants (e.g. diaphyseal spacers, arthodeses, nails etc.), even if it was a large implant used for treatment of a metastatic lesion close to a joint, were not included in the study.

**Table:** Descriptive data for 130 patients who had 140 joint replacements because of metastatic bone disease during the period 2003–2008

No. of patients	130
Female/male	76/54
Age at surgery, years mean (range)	64 (30–85)
Primary tumor site	
breast	31
lung	20
kidney	16
prostate	15
myeloma	12
unknown	9
lymphoma	5
malignant melanoma	4
bladder	4
sarcoma	4
other	10
No. of operations	140
Major bone resection	
yes/no	103/37
Joints replaced	
hip	105
shoulder	16
knee (distal femur)	14
elbow	5
Type of bone lesion	
pathological fracture	101
osteolytic lesion	38
sclerotic lesion	1

Breast cancer (n = 31), lung cancer (n = 20), and kidney cancer (n = 16) were the predominant causes of the skeletal metastases leading to joint replacement (Table). 36 patients had only 1 bony metastasis, 21 patients had 2 or 3 metastases, 70 patients had multiple metastases, and in 3 patients the data regarding the number of metastases were missing.

The study was approved by the Danish Data Protection Agency (no. 2008-41-2819) and the Danish Health and Medicines Authority (no. 7-505-29-1642/1). The study was evaluated by the Regional Scientific Ethical Committee of the Capital Region of Denmark (no. H-3-2010-130) and it was not considered to be a notifiable study.

### Operation

The bony lesions were located in the lower extremities in 119 cases (proximal femur: 105; distal femur: 14) and in the upper extremities in 21 cases (distal humerus: 5; shoulder: 16). The indication for surgery of the individual joints was: a pathological fracture (n = 101), an osteolytic lesion (n = 38), or a sclerotic lesion (n = 1) (Table). 3 patients had 2 joint replacements performed in different anatomical locations as a 1-stage procedure and 1 patient had 3 such joint replacements. 5 patients had more than 1 joint replacement performed on different days during the study period. Major bone resection—defined as resection through or below the lesser trochanter at the hip, above the femoral condyles at the knee, below the humeral head, and above the humeral condyles at the elbow—was performed in 103 operations.

**Figure 1.**
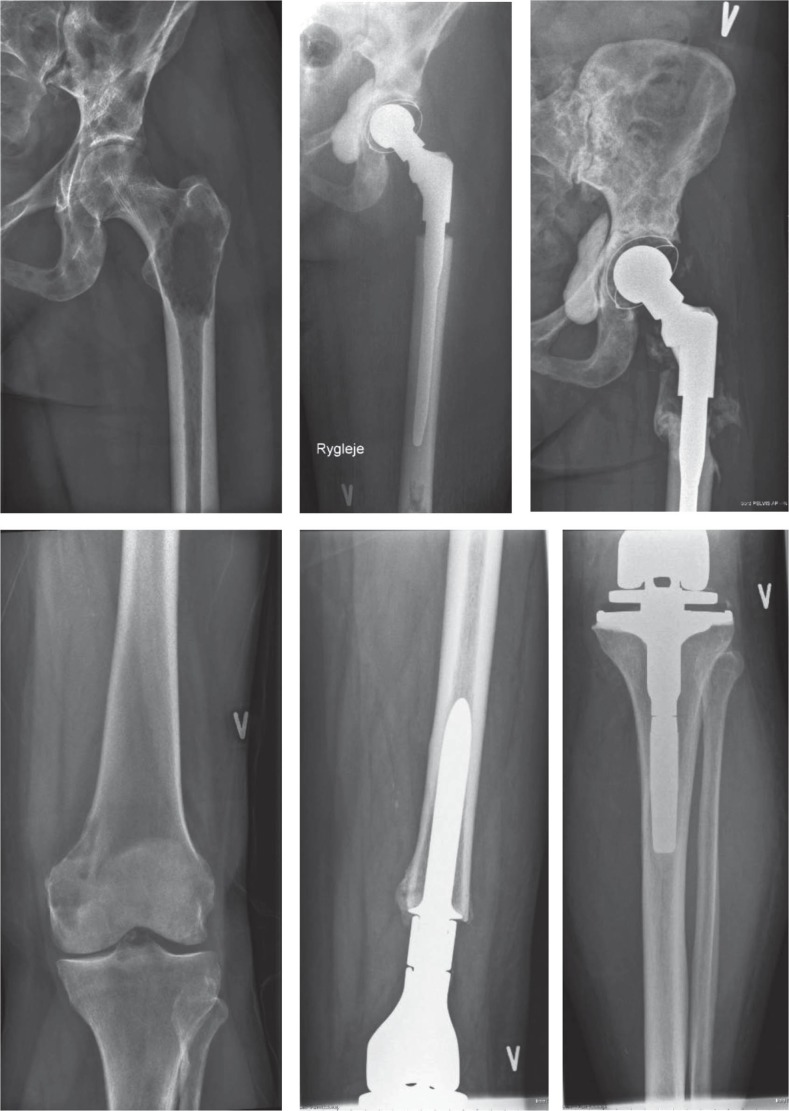
Upper panels. A patient suffering from breast cancer and left hip pain because of multiple osteolytic metastases of the left hip and acetabular region (left panel); postoperatively, after resection of the proximal femur and insertion of a total hip replacement using an MP reconstruction hip stem (middle panel) and status 4 years later (right panel). Lower panels. A patient with previous cancer of the bladder, suffering from knee pain because of a solitary metastasis of the medial femoral condyle (left panel); status 4 months postoperatively after resection of the distal femur and reconstruction with a GMRS prosthesis (middle and right panels).

The following types of implants were used for reconstruction of the hip joint and surrounding bone defects: Bimetric stems for primary total hip replacement (n = 5), medium or long stem revision prostheses (n = 97) (i.e. Bimetric revision stems (n = 55), MP reconstruction hip stems (n = 22) (Figure 1), RX 90 prostheses (n = 14), long Kent hip stems (n = 5), and long Lubinus revision stem (n = 1)), and modular tumor prostheses (n = 3) (HMRS, GMRS, Mega C). With the exception of 3 hips that received bipolar heads, all hips (with very few exceptions) received a cemented Lubinus cup. At the knee, the following prostheses were used: GMRS tumor prostheses (n = 7) (Figure 1), Endorotational knee (n = 6), and Mega C (n = 1). For shoulder and elbow replacements, the following prostheses were used: Bigliani/Flatow (n = 15), HMRS tumor prosthesis (n = 1), and Coonrad/Moorey (n = 5).

Average blood loss during surgery ((n = 134), missing data in 4 patients (6 operations)) was 1.3 (0.1–7) L.

### Data and statistics

Clinical data on complications and reoperations after the joint replacement operation were extracted from the patient files. To supplement these data and to compensate for a lack of data in patients with short follow-up in our hospital or missing patient files (n = 4), data were also extracted from the Danish National Registry of Patients on March 29, 2011. Survival data were extracted from the Danish Central Civil Register on March 29, 2011. Thus, the follow-up for both survival and clinical data was at least 28 months or until death, giving a mean follow-up time of 17 (0–96) months from the operation.

Kaplan-Meier survival analysis was used for estimation of the probability of patient survival, the probability of surgical removal or replacement of 1 or all of the prosthetic components anchored to the bone, and the probability of avoiding all kinds of surgery related to the implanted prosthesis. If a patient was treated for more than 1 metastasis during the study period, patient survival was calculated from the time of the first joint replacement only. To be able to compare survival to a wide range of previous studies, we also calculated a median value for survival.

We used IBM SPSS software (version 19) for most of the statistical calculations. Calculation of 95% confidence intervals (CIs) for the survival data was done in Microsoft Excel 2010 using Greenwood’s formula for calculation of standard error.

## Results

**Figure 2.**
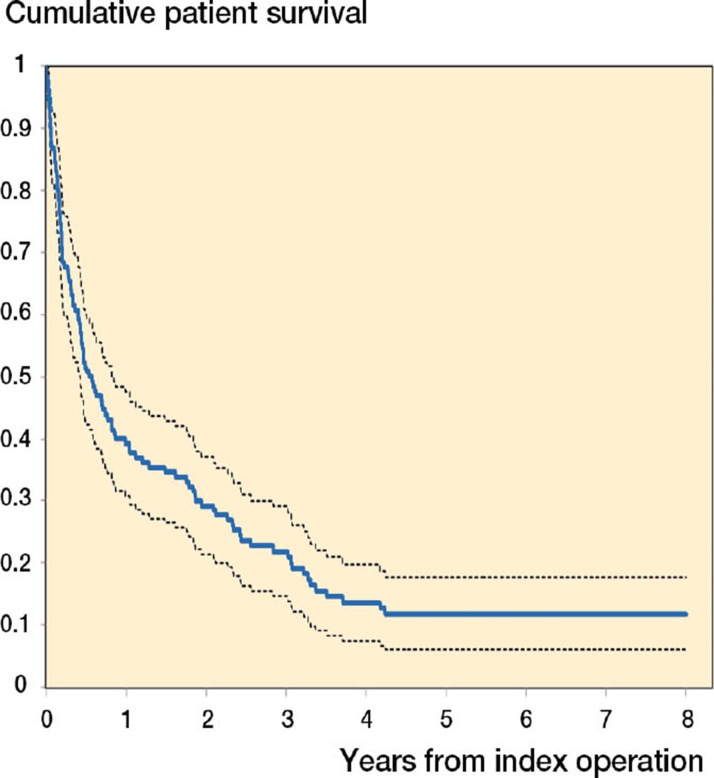
Cumulative survival rate (solid line) and 95% confidence interval (dotted lines) for 130 patients who had 1 or more joint replacements because of metastatic bone disease during the period 2003–2008.

The calculated probability of patient survival was 51% (CI: 42–59), 39% (CI: 31–48), 29% (CI: 21–37), and 22% (CI: 15–29) at 6 months and 1, 2, and 3 years of follow-up (Figure 2), and the median survival time was 7 (0.03–96) months. 17 patients died in the early postoperative period (within 30 days after operation) and 4 of these patients died of an illness that could be traced directly to the operation or to the period of time under general anesthesia: cardiac arrest (n = 2), pulmonary embolism (n = 1), and severe hypotension (n = 1). 25 patients were long-term survivors beyond 3 years, and the underlying disease in these patients was: hematological disease (n = 12: 8 myelomatosis and 4 lymphoma), breast cancer (n = 7), prostate cancer (n = 2), kidney cancer (n = 2), lung cancer (n = 1), and uterine leiomyosarcoma (n = 1).

The following 15 complications directly related to the joint replacement operations were seen: hip dislocations 2–5 times (n = 8), deep infection (n = 3), peroneal palsy (n = 2), a shoulder prosthesis penetrating the skin more than 4 and a half years after implantation (n = 1), and an early disassembly of an elbow joint prosthesis (n = 1). The complications led to additional surgery in 7 of the patients (9 operations). The patient with disassembly of an elbow joint prosthesis had a mechanical component replaced 2 months after surgery, and the same patient later developed deep infection leading to removal of the prosthesis 1 year after the initial operation. Because of early recurrent hip dislocation 2 hip replacement patients were reoperated on 2–3 months after the index operation with implantation of a device that made the cup constraint. Another hip patient had the cup revised 7 months after implantation of the prosthesis due to early recurrent dislocation of the hip (5 times within 6 months after the initial operation). 1 patient had soft tissue revision due to deep infection 8 months and 2.3 years after implantation of an MP hip prosthesis. The patient had long-term antibiotic treatment and the prosthesis was never removed. 1 knee patient had 1-stage revision surgery with replacement of a Mega C distal femur tumor prosthesis due to deep infection 1.3 years after implantation. Finally, the patient who had a shoulder replacement penetrating the skin had the prosthesis removed.

**Figure 3.**
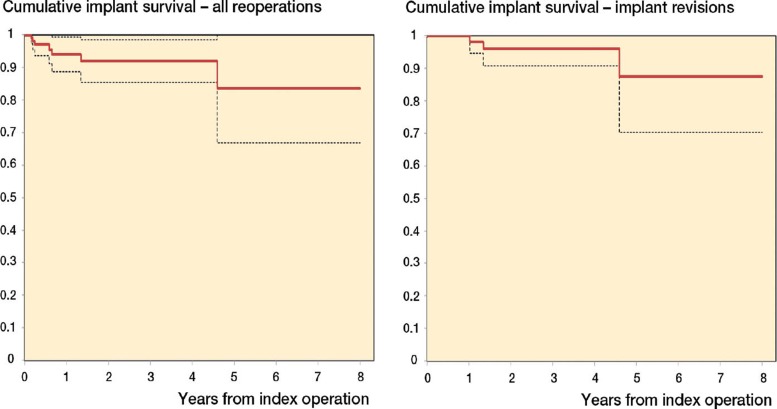
Cumulative survival rate (solid line) and 95% confidence limits (dotted line) for all 140 joint replacements inserted because of metastatic bone disease during the period 2003–2008. The probability of survival was calculated with either all kinds of surgery of the affected joint (left panel) or removal of at least 1 prosthetic component anchored to bone (right panel) as endpoint in the Kaplan-Meier analysis.

The probability of avoiding all kinds of surgery related to the implanted prosthesis was 94% (CI: 89–99) after 1 year, 92% (CI: 85–98) after 2 years, and 84% (CI: 67–100) after 5 years (Figure 3 left). When only removal or replacement of components anchored to the bone was considered an event in the survival analysis, the probability of prosthesis survival was 100%, 96% (CI: 91–100), and 87% (CI: 70–100) after 1, 2, and 5 years (Figure 3 right).

## Discussion

Several studies have evaluated patient survival following operative treatment of metastatic bone disease, but few have involved 100 or more patients with precise information of patient survival data (Bauer and Wedin 1995, Wedin et al. 1999, Hansen et al. 2004, Camnasio et al. 2008, Chandrasekar et al. 2008, Sarahrudi et al. 2009, Harvey et al. 2012, Wedin et al. 2012). With the exception of one of them (Chandrasekar et al. 2008) where it was not obvious if patients with malignant hematological diseases were included, these studies all had an almost identical distribution of the various primary tumors to what was seen in the present study (Bauer and Wedin 1995, Wedin et al. 1999, Hansen et al. 2004, Camnasio et al. 2008, Sarahrudi et al. 2009, Harvey et al. 2012, Wedin et al. 2012). Some of the largest studies were registry studies from the Scandinavian Sarcoma Group (Hansen et al. 2004, Wedin et al. 2012) and in the study by Hansen et al., patient survival after operative treatment of metastases of the extremities and pelvis in 460 patients was evaluated. These authors found a 1-year survival of 39% in a material consisting of an almost equal number of osteosyntheses and joint replacements, and operation method was not related to survival. Wedin et al. (2012) presented survival data from 208 patients who were treated operatively for bone metastases of the upper extremities and they found a 1-year survival of 40% in a material dominated by lesions treated by osteosythesis (mainly intramedullar nails (n = 148)) and with only 35 joint replacements of the shoulder joint.

Bauer and Wedin (1995) evaluated the survival in 153 patients who were treated surgically (with no information regarding the type of treatment) for an extremity metastasis, and they found a 1-year survival of 31%. Wedin et al. (1999) reviewed 192 patients who underwent 228 operations for metastatic lesions of a long bone (54 joint replacements and 162 ostesynthesis), and they found a 1-year survival of 30%. Harvey et al. (2012) retrospectively evaluated the survival of 158 patients treated with either an intramedullar nail (n = 46) or a joint replacement (n = 113) because of a metastatic lesion of the proximal third of the femur, and a 1-year survival of 51% was found. Camnesio et al. (2008) evaluated patient survival in 154 patients treated with bone resection and joint replacement because of bone metastases over a 13-year period, and they found a 1-year survival of 70%. Chandrasekar et al. (2008) evaluated patient survival following operation with bone resection and reconstruction of the proximal femur with a modular tumor prosthesis in 100 consecutive patients who were operated on during a period of 6 years, and these authors found a 1 year survival of 35%.

The 1-year survival of 39% that we found is similar to that (30–40%) found in previous Scandinavian studies reporting data from patients treated with a joint replacement or various types of osteosynthetic device (Bauer and Wedin 1995, Wedin et al. 1999, 2012, Hansen et al. 2004). 1-year survival rates ranging from 17% to 70% have been found in various studies (Bauer and Wedin 1995, Wedin et al. 1999, 2012, Hansen et al. 2004, Camnasio et al. 2008, Chandrasekar et al. 2008, Sarahrudi et al. 2009, Harvey et al. 2012). In studies evaluating survival in patients treated solely with joint replacement, the 1-year survival was 35–70% (Camnasio et al. 2008, Chandrasekar et al. 2008). Thus, no data from our study or from previous studies indicate a higher mortality rate in patients treated with joint replacements than in those treated with osteosynthetic devices, and the very different survival rates between the studies are probably more an expression of different criteria for selecting patients for surgical treatment than an effect of the treatment itself. Furthermore, when comparing survival of the patients in the present study to that in patients treated with radiotherapy alone (Steenland et al. 1999), the median survival is the same, thus indicating that undergoing surgery for treatment of metastatic bony lesions does not reduce life length compared to nonoperative treatment such as radiotherapy.

In patients with metastatic bone disease, it is also important that the surgical treatment is uncomplicated with only minor risk of reoperation, especially when taking the short expected patient survival into consideration. In our material, the infection rate after the index operation was 2% (2/140 joint replacements), which is just slightly above the rate in primary joint replacements. However, the real infection rate in our patients was probably underestimated because we cannot rule out the possibility that a few patients with very poor health could have been given long-term antibiotic treatment in a local hospital because of a suspected deep infection, without our knowledge. Furthermore, the high mortality of the patients also reduces the probability that a late infection with low virulent bacteria would become a clinical problem.

Recurrent hip dislocation was seen in 8% (8/105 hips) and peroneal palsy in 2% (2/105 hips). The rates of peroneal palsy and of hip dislocation were higher than that in primary total hip replacement, and were closer to the level in revision total hip replacement (Garcia-Cimbrelo and Munuera 1992, Schmalzried et al. 1997). This is not surprising, because the operations in our patients were often similar to revision joint replacements regarding duration and magnitude of the surgery. Furthermore, in several of the operations we performed a major bone resection sometimes combined with removal of a soft tissue tumor component. It was policy in our department to use total hip replacement, while in many clinics a hemiarthroplasty is preferred when treating metastatic bone disease at the hip. The use of hemiarthroplasty in our study population could most likely have reduced the risk of hip dislocation and the risk of early reoperation in some patients. However, later acetabular cartilage degeneration may necessitate later conversion to total hip arthroplaty in long-term survivors if hemiarthroplasty is used (Kim et al. 2012).

Due to poor survival of the patients, the implant survival in our study was difficult to compare with the level seen in primary joint replacements, but not surprisingly it was far below the implant survival found in registry studies evaluating implant survival after primary knee replacement or hip replacement (Hailer et al. 2010, Paxton et al. 2011). However, in studies comparing implant survival in patients operated with osteosynthesis and in those operated with joint replacement, the joint replacement group has shown a lower mechanical failure rate and a higher rate of implant survivorship (Wedin et al. 1999, Harvey et al. 2012).

The design of our study allowed more than 1 observation in each patient when evaluating implant complications and survival, which could have introduced bias (Bryant et al. 2006). However, it happened in only 8 patients, who either had 2 or 3 joint replacements as a 1-stage procedure or had 2 or 3 joint replacements on different days during the study period. However, in the patient survival analysis this bias was not allowed, since each patient could only be entered into the survival analysis at the time of the first joint replacement operation in the study period.

In conclusion, joint replacement operations because of metastatic bone disease did not appear to give a poorer patient survival rate than other types of surgical treatment. However, this conclusion relies only on a comparison between the results of various descriptive studies that have obviously had different criteria for allocation of the patients to joint replacement or osteosynthesis; no prospective randomized studies dealing with this problem have been published. The reoperation and complication rate was low, but it was higher than in primary joint replacement and closer to what is seen after revision joint replacement surgery.
